# Assessing the Quality of Life of Parents of Children With Disabilities Using WHOQoL BREF During COVID-19 Pandemic

**DOI:** 10.3389/fresc.2021.708657

**Published:** 2021-08-13

**Authors:** Usman Ali, Vardah Bharuchi, Nasreen Gulzar Ali, Sidra K. Jafri

**Affiliations:** Department of Pediatric and Child Health, Aga Khan University Hospital, Karachi, Pakistan

**Keywords:** quality of life, parents, children with developmental disabilities, COVID-19, WHOQoL

## Abstract

**Background:** Caring for a child with disabilities is a challenging journey, as the parents must meet greater demands when compared with the parents of children without disabilities. Looking after a child with disablities requires additional financial, social, emotional, and physical resources. Coronavirus 2019 (COVID-19) pandemic has made this even more challenging and impacted the quality of life of parents of children with disabilities.

**Methods:** The study was an analytical cross-sectional design with two comparison groups: parents of children with developmental disabilities and parents of children without disabilities. The Urdu version of the WHO Quality of Life Measure Abbreviated version (WHOQOL-BREF) was used to measure the quality of life (QoL) among parents. Sociodemographic data were also obtained from the parents.

**Results:** Parents of children with disabilities had lower overall scores when different domains of QoL were considered (physical health, psychological health, social relationships, and environment) using WHOQoL-BREF. Statistically significant differences were observed in the physical and environmental domains of parental QoL.

## Introduction

Caring for a child with developmental disabilities is a challenging journey, as the parents must meet greater demands when compared with the parents of children without disabilities. Such parents require additional financial, social, emotional, and physical resources, which may conflict with the competing needs of the other family members. This life-long journey of parents navigating through the medical, developmental, and educational interventions in addition to caregiving responsibilities affects their quality of life (QoL) (1).

Quality of life refers to the overall well-being of individuals in multiple aspects of life; however, its definition has evolved through time (2). It is a multifaceted concept that comprises perceived psychological, social, emotional, and physical functioning of an individual and is often used to examine the well-being of and burdens in families with neurodevelopmental disorders. WHO defines QoL as “an individual's perception of their position in life in the context of the culture and value systems in which they live and in relation to their goals, expectations, standards and concerns” (3). QoL is therefore affected by physical health, psychological state, personal beliefs, social relationships, and relationship with the environment of a person (4).

Quality of life is a difficult concept to define and measure, and there are over a thousand instruments designed to measure the QoL (5). The WHO Quality of Life (WHOQOL)-BREF instrument was developed by the WHO as a result of a global project based on a cross-culturally sensitive concept and is considered appropriate for use across different nationalities (6–8). WHOQoL-BREF measures four domains of QoL, physical health, psychological health, social relationships, and environment, through a set of 24 items (2). In addition to cross-culture applicability, this tool has good to excellent reliability and validity (6–10).

Understanding the different aspects of QoL is essential for parents having a child with developmental disabilities to guide the healthcare workers and policymakers to have a better insight into the struggle faced by parents for planning and implementing different interventions (11).

COVID-19 pandemic, an unexpected catastrophe, has overwhelmed the capacity of the healthcare systems and affected nearly all facets of QoL for the general population. COVID-19 has major ramifications for global health, including the disability community. It has also caused school closures, limited availability of support services, and income loss (12, 13). Children with severe disabilities are physically more demanding and their parents are expected to experience increased caregiver burden during this unprecedented pandemic.

This study aimed to assess the QoL in parents of children with developmental disabilities when compared to typically developing peers in the four domains, namely physical health, psychological health, social relationships, and environment, and overall QoL during the Covid 19 pandemic. Additionally, we aimed to compare the WHOQoL-BREF scores between different developmental disabilities.

## Materials and Methods

This analytical cross-sectional study with a comparison group was performed at the Pediatric Outpatient Department at the Aga Khan University Hospital, Karachi, Pakistan, after approval by the Institutional Ethics Review Committee (ERC-2020-5476-14718). Parents of children (2–18 years) with developmental disabilities, such as cerebral palsy (CP), autism spectrum disorder (ASD), global developmental delay (GDD), intellectual disability (ID), attention deficit hyperactivity disorder (ADHD), speech delays, learning disabilities (LD), or any syndrome or condition with any of these conditions and receiving services, were included in the study group.

Parents of typically developing children (aged 2–18 years) visiting the Pediatric Outpatient Department (OPD) for routine check-ups or common pediatric problems without any underlying disability were included in the comparison group.

Parents were excluded if they did not understand Urdu or could not fill out the questionnaire.

Parents were included using a consecutive sampling technique. GPower software was utilized to calculate the sample size using the difference in parental QoL of children with disabilities and those of children without disabilities. Literature reports the effective size of the difference in QoL among the two groups is in the range of 0.3–0.4 in various domains (14). Being a ow-middle-income country (LMIC), we anticipated higher intensity of the effect in our population. A sample size of minimum of 100 children per group was required at 80% power and Cronbach's alpha of 0.05 to detect the difference of at least 0.7 points with SD of 2.2; however, we inflated our sample size to 150 participants in each group.

Study participants were administered a questionnaire that contained demographic questions followed by WHO-QOL-BREF-Urdu. The demographic information included the parental age, parent gender, family status(nuclear/joint), marital status (married/divorced/separated/widowed), education level of both parents (no education, can read or write, primary (Grade 5), secondary (Grade 10), graduate, postgraduate), work status of both parents (full-time, part-time, unemployed), monthly family income (<PKRs25K, PKRs25–50K, PKRs50–100K, > PKRs100K), the health status of the parents (healthy/known comorbidity), and age, gender, and diagnosis of the children.

The forms were collected by one of the co-investigators, and the participants were assigned unique codes to protect their identity and to maintain confidentiality.

World Health Organization Quality of Life-BREF questionnaire contained 24 items covering four domains: physical health, psychological health, social relationships, and environment (10). The mean score of items within each domain was used to calculate the mean domain score, after which the mean scores were multiplied by four to make domain scores comparable with the scores used in the WHOQOL-100, as per the instructions of the measure used. Domain scores were scaled in a positive direction with a score range of 0–100 with higher scores denoting higher QoL.

### Data Analysis

Data were analyzed using IBM SPSS version 23. The sociodemographic variables were analyzed using descriptive statistics. The Chi-square test and the Mann–Whitney *U*-tests were carried out to observe the statistical differences between the sociodemographic variables of the parents of children with disabilities and those of children without disabilities. An independent samples *t*-test was used to analyze the difference between the scores of parents of typically developing children and parents of children with disabilities on the subdomains of WHOQoL-BREF. A multiple regression was carried out to evaluate which sociodemographic variables can significantly predict the scores of the subdomains and the overall score of WHOQoL-BREF.

## Results

A total of 301 participants were recruited for the study, out of which 151 participants were the parents of children without disabilities and 150 participants were the parents of children with developmental disabilities. The demographic data of the participants are given in Table 1.

**Table 1:** Sociodemographic data of parents with children without developmental disabilities (group A) and children with developmental disabilities (group B).

	**Group A *n* (%)**	**Group B *n* (%)**
Total no. of parents	151 (50.2)	150 (49.8)
**Age**		
15–25 years	15 (9.93)	6 (4.0)
26–30 years	53 (35.10)	56 (37.33)
31–35 years	52 (34.44)	56 (37.33)
36–45 years	20 (13.25)	26 (17.33)
more than 45 years	11 (7.28)	6 (4.0)
**Gender**		
Male	49 (32.45)	60 (40.00)
Female	102 (67.55)	89 (58.33)
**Marital status**		
Married	149 (98.68)	149 (99.33)
Divorced	0	1 (0.67)
Widowed	0	0
**Family status**		
Nuclear	60 (39.74)	73 (48.76)
Joint	91 (61.26)	77 (51.33)
**Mother's educational status**		
No education	3 (1.99)	5 (3.33)
Can read or write	7 (4.64)	6 (4.00)
Primary	11 (7.28)	9 (6.00)
Secondary	38 (25.17)	28 (18.67)
Graduate	68 (45.03)	79 (52.67)
Post-graduate	24 (15.89)	23 (15.33)
F**ather's educational status**		
No education	0	3 (2.00)
Can read or write	2 (1.32)	4 (2.67)
Primary	7 (4.64)	9 (6.00)
Secondary	31 (20.53)	31 (20.67)
Graduate	67 (44.37)	60 (40.00)
Post-graduate	41 (27.15)	43 (28.67)
**Mother's work status**		
Full-time	18 (11.92)	26 (17.33)
Part-time	7 (4.64)	7 (4.67)
Unemployed/stay at home	125 (82.78)	117 (78)
**Father's work status**		
Full-time	136 (90.07)	135 (90.00)
Part-time	9 (5.96)	12 (8.00)
Unemployed/stay-at-home	4 (2.56)	1 (0.67)
**Monthly family income, PKR**		
<25,000	9 (5.96)	8 (5.33)
26,000–50,000	30 (19.87)	40 (26.67)
50,000–s100,000	60 (39.74)	56 (37.33)
>100,000	52 (34.44)	46 (30.67)
**Mother's health status**		
Healthy	137 (90.73)	144 (96.00)
Known comorbid	14 (9.27)	6 (4.00)
**Father's health status**		
Healthy	140 (92.72)	136 (90.67)
Known comorbid	9 (5.96)	14 (9.33)

*Missing data: Group A missing: marital status: 2; father education status: 3; work status of mother: 1; work status of father: 2; health status of father: 2; gender of child: 1, Group B missing gender: 11; work status of father: 2. Total missing: type: 2; diagnosis of child: 2*.

Children with different disabilities were identified. The common disabilities observed were ASD, GDD, and CP. The details are depicted in Table 2.

**Table 2:** Demographic table of parents with children without developmental disabilities (group A) and children with developmental disabilities (group B).

**Variable**	**Group A *n* (%)**	**Group B *n* (%)**
**Age of the parent**		
<5 years	104 (68.9)	96 (64.9)
5– <12 years	43 (28.5)	45 (30.4)
12–18 years	4 (2.6)	7 (4.7)
**Diagnosis**		
Autism spectrum disorder	0 (0)	74 (50.0)
Attention deficit hyperactivity disorder	0 (0)	9 (6.1)
Cerebral palsy	0 (0)	23 (15.5)
Global developmental delay	0 (0)	27 (18.2)
Learning disability	0 (0)	3 (2.1)
Speech delay	0 (0)	12 (8.1)

The Chi-square test was carried out to see the statistical differences between parents of children without disabilities (group A) and parents of children with disabilities (group B) in terms of gender, education level, work status, and marital status. No significant differences were observed (Table 3).

**Table 3:** Comparison of parents with children without developmental disabilities (group A) and children with developmental disabilities (group B).

**Variable**	**Value**	*p* ^*^
Gender	2.247	0.134
Education level of mother	3.034	0.695
Education level of father	4.437	0.488
Work status of mother	1.462	0.481
Work status of father	1.985	0.371
Marital status	1.010	0.315

* *2-tailed level of significance: p < 0.05*.

The Mann–Whitney *U*-test was carried out between parents of non-disabled children (group A) and parents of children with disabilities children (group B). No significant differences were observed between the scores of the two groups.

An independent samples *T*-test was used to analyze the difference between the scores of parents of children without disabilities and parents of children with disabilities. Parents of children with disabilities scored *lower* overall when different domains of QoL were considered (physical health, psychological health, social relationships, and environment) (Tables 4 and 5). Significant differences were observed for physical health in parents of children without disabilities (M = 66.09, SD = 12.629) and children with disabilities (M = 61.80, SD = 15.652); *t* (285.945) = 2.609, *p* = 0.010. Similarly, significant differences were also observed for social relationships in parents of children without disabilities (M = 73.40, SD = 16.272) and those of children with disabilities (M = 68.37, SD = 18.368); *t* (297) = 2.509, *p* = 0.013.

**Table 4:** Comparison between the mean scores of parents of children without developmental disabilities (group A) and children with developmental disabilities (group B).

**Item**	***n***	**Mean rank**	**Z**	*p* ^*^
Age of the parent			−0.812	0.417
Group A	151	146.18		
Group B	148	153.90		
Monthly family income			−929	0.353
Group A	151	154.35		
Group B	148	145.56		
Age of the child			−0.828	0.408
Group A	151	146.63		
Group B	148	153.44		

* *2-tailed. Level of significance: p < 0.05*.

**Table 5:** Comparison of quality of life (QoL) between parents of children without developmental disabilities (group A) and children with disabilities (group B).

**Variable**	***n***	**Mean**	**SD**	**T**	*p* ^*^
Domain 1: physical health				2.614	0.009
Group A	151	66.09	12.629		
Group B	148	61.80	15.652		
Domain 2: psychological health				1.333	0.183
Group A	151	65.51	14.834		
Group B	148	63.25	14.475		
Domain 3: social relationships				2.506	0.013
Group A	151	73.40	16.272		
Group B	148	68.37	18.368		
Domain 4: environment				2.696	0.007
Group A	151	71.99	13.510		
Group B	148	67.79	13.401		
WHO QOL (BREF)				2.96	0.003
	151	89.75	10.35		
	148	85.81	12.56		

* *2-tailed. Level of significance: p < 0.05. SD, standard deviation*.

Significant differences were observed for environment between parents of children without disabilities (M = 71.99, SD = 13.510) and those of children with disabilities (M = 67.79, SD = 13.401); *t* (296.957) = 2.696, *p* = 0.007. No significant difference was observed in psychological health between parents of children without disabilities (M = 65.51, SD = 14.834) and those of children with disabilities (M = 63.25, SD = 14.475); *t* (286.994) = 1.333, *p* = 0.183. The overall score of WHOQoL-BREF also showed a significant difference between children without disabilities (M = 89.75, SD = 10.35) and children with disabilities (M = 85.81, SD = 12.56); *t* (284.41) = 2.96, *p* = 0.003.

A multiple regression analysis was carried out to identify potential risk factors for QoL in parents. It was done to predict the score of physical health of parents from the domains of diagnosis of education status, health status, work status, gender of parents, monthly income, family status, gender, age, and diagnosis of children (Table 6). These variables statistically significantly predicted the score on the physical health domain of WHOQoL-BREF (14, 276) = 1.886, *p* = 0.028, with an R^2^ of.087. However, the health status variable of the father (B = −8.649, *p* = 0.016) contributed statistically significantly to the prediction.

**Table 6:** Summary of a multiple regression analysis of physical health in WHOQoL-BREF.

	**Physical health**		
	**B**	**SE B**	**B**
Constant	83.113	16.870	
Type	−8.953	4.911	−0.311
Age	0.623	1.010	0.043
Gender	−0.401	1.795	−0.013
Family status	1.024	1.780	0.035
Education status of mother	1.252	0.996	0.099
Education status of father	0.558	1.095	0.040
Work status of mother	1.279	1.357	0.062
Work status of father	−3.472	2.693	−0.082
Monthly income	−0.820	1.062	−0.050
Health status of mother	0.610	3.684	0.010
Health status of father	−8.649	3.584	−0.159
Age of children	−0.587	1.761	−0.023
Gender of child	0.022	1.735	0.001
Diagnosis of child	−0.770	0.709	−0.184
R-squared	0.087		
Adjusted R- square	0.041		
F for change in R square	1.886^*^		
No. of observations	290		

* *Level of significance: p < 0.05. SE, standard error*.

A multiple regression analysis was also carried out to predict the score of psychological health of parents from the domains of education status, health status, work status, gender of parents, monthly income, family status, gender, age, and diagnosis of children (Table 7). These variables showed statistically significantly predicted the score on the psychological health domain of WHOQoL, *F* (14, 276) = 2.890, *p* = 0.000, with an R^2^ of.128. However, the health status variable of the father (B = −15.787, *p* = 0.000) contributed statistically significantly to the prediction.

**Table 7:** Summary of a multiple regression analysis of psychological health in WHOQoL-BREF.

	**Psychological health**		
	**B**	**SE B**	**B**
Constant	84.921	16.539	
Type	−2.192	4.815	−0.076
Age	1.388	0.990	0.095
Gender	−1.775	1.760	−0.059
Family status	0.360	1.745	0.012
Education status of mother	1.214	0.976	0.095
Education status of father	−0.156	1.074	−0.011
Work status of mother	0.299	1.330	0.015
Work status of father	−0.426	2.640	−0.010
Monthly income	−0.955	1.041	−0.059
Health status of mother	−3.008	3.611	−0.052
Health status of father	−15.787	3.514	−0.289
Age of children	−2.708	1.727	−0.104
Gender of child	2.263	1.701	0.078
Diagnosis of child	−0.015	0.695	−0.004
R-squared	0.128		
Adjusted R- square	0.084		
F for change in R square	2.890^*^		
No. of observations	290		

* *Level of significance: p < 0.05. SE, standard error*.

A multiple regression analysis was carried out to predict the score of social relationships of parents from the domains of education status, health status, work status, gender of parents, monthly income, family status, gender, age, and diagnosis of children (Table 8). These variables statistically significantly predicted the score on the social relationships domain of WHOQoL, *F* (14, 276) = 1.835, *p* = 0.034, with an R^2^ of.085. However, no single variable contributed significantly to the prediction of the model.

**Table 8:** Summary of a multiple regression analysis of social relationships in WHOQoL-BREF.

	**Social relationships**		
	**B**	**SE B**	**β**
Constant	59.603	20.571	
Type	3.741	5.989	0.107
Age	−1.757	1.232	−0.099
Gender	2.688	2.189	0.074
Family status	0.725	2.170	0.021
Education status of mother	−0.088	1.214	−0.006
Education status of father	0.076	1.335	0.004
Work status of mother	1.940	1.654	0.078
Work status of father	−1.131	3.283	−0.022
Monthly income	1.427	1.295	0.072
Health status of mother	−3.909	4.492	−0.055
Health status of father	−8.390	4.371	−0.127
Age of children	1.961	2.148	0.062
Gender of child	0.107	2.115	−0.003
Diagnosis of child	1.287	0.865	0.253
R-squared	0.085		
Adjusted R- square	0.039		
F for change in R square	1.835^*^		
No. of observations	290		

* *Level of significance: p < 0.05. SE, standard error*.

Another multiple regression analysis was carried out to predict the score of the environment on parents from the domains of education status, health status, work status, gender of parents, monthly income, family status, gender, age, and diagnosis of children (Table 9). These variables statistically significantly predicted the score on the environment domain of WHOQoL, *s* (14, 276) = 4.654, *p* = 0.000, except for the age of parents variable (*p* = 0.044), with an R^2^ of.185. However, the education status of the mother (B = 2.495, *p* = 0.005), work status of the mother (B = 2.680, *p* = 0.027), and monthly income (B = 2.833, *p* = 0.003) variables contributed statistically significantly to the prediction.

**Table 9:** Summary of a multiple regression analysis of the environment in WHOQoL-BREF.

	**Environment**		
	**B**	**SE B**	**B**
Constant	35.784	14.960	
Type	−0.739	4.355	−0.027
Age	−1.692	0.869	−0.118
Gender	2.820	1.592	0.100
Family status	0.034	1.578	0.001
Education status of mother	2.495	0.883	0.209
Education status of father	0.200	0.971	0.015
Work status of mother	2.680	1.203	0.139
Work status of father	3.261	2.388	0.081
Monthly income	2833	0.941	0.185
Health status of mother	6.80	3.267	0.012
Health status of father	−4.426	3.178	−0.086
Age of children	1.793	1.562	0.073
Gender of child	1.915	1.538	0.070
Diagnosis of child	0.432	0.629	0.110
R-squared	0.191		
Adjusted R- square	0.150		
F for change in R square	4.654^*^		
No. of observations	290		

* *Level of significance: p < 0.05. SE, standard error*.

A multiple regression was carried out to predict the overall general score of parents on WHOQoL from the domains of education status, health status, work status, gender of parents, monthly income, family status, gender, age, and diagnosis of children (Table 10). These variables statistically significantly predicted the overall score of WHOQoL, *F* (14, 276) = 3.535, *p* = 0.000, with an R^2^ of.152. However, the education status of the mother variable (B = 1.727, *p* = 0.027) contributed statistically significantly to the prediction.

**Table 10:** Summary of a multiple regression analysis of overall WHOQoL-BREF.

	**Total raw score**		
	**B**	**SE B**	**β**
Constant	75.890	13.147	
Type	−1.654	3.827	−0.071
Age	−0.508	0.787	−0.043
Gender	2.074	1.399	0.086
Family status	0.351	1.387	0.015
Education status of mother	1.727	0.776	0.168
Education status of father	0.957	0.853	0.084
Work status of mother	2.046	1.057	0.123
Work status of father	−1.762	2.098	−0.051
Monthly income	0.461	0.827	0.035
Health status of mother	−1.189	2.871	−0.025
Health status of father	−5.306	2.793	−0.121
Age of children	−0.125	1.373	−0.006
Gender of child	−0.011	1.352	0.000
Diagnosis of child	0.284	0.553	0.084
R-squared	0.152		
Adjusted R- square	0.109		
F for change in R square	3.535^*^		
No. of observations	290		

* *Level of significance: p < 0.05. SE, standard error*.

### Summary of Key Results

- No statistically significant difference was observed between parents of children without disabilities (group A) and parents of children with developmental disabilities (group B) in terms of gender, education level, work status, and marital status.
- No significant difference was observed between parents of children without disabilities (group A) and parents of children with developmental disabilities (group B) in terms of parental age, monthly income, and age of children.
- Parents of children with disabilities had a lower overall score when different domains of QoL were considered (i.e., physical health, psychological health, social relationships, environment, and overall QoL). Significant differences were observed for physical health, social relationships, environment, and overall QoL. No significant difference was observed for psychological health.

Multiple regression analysis results:

- The health status of the father provided a statistically significant score to the predictive model on the physical health subdomain of WHOQoL-BREF.
- To predict the score of psychological health of parents, the health status of the father added statistical significance to the prediction.
- To predict the score of social relationships of parents, no single variable added statistical significance to the prediction.
- To predict the score of the environment of parents, the education status of the mother, the work status of the mother, and the monthly income added statistical significance to the prediction.
- To predict the overall general score of parents on WHOQoL, the education status of the mother significantly predicted the score on WHOQoL.

## Discussion

Global estimates demonstrate that 15% of the world population is affected by some form of disability (15). Among these, between 110 and 190 million individuals have significant functional limitations and participation restrictions (16). No estimates based on actual measurement of the number of children with disabilities are available; however, a UK study estimated the national prevalence of childhood disability at 7.3% of the population with the highest prevalence of childhood disability seen in the poorest income quintile (17). The epidemiology of disability in Pakistan is limited; however, the prevalence of childhood disability was found to be 5.5 out of 1,000 in rural Sindh (18). According to UNICEF, in developing countries like Pakistan, children at an early age are exposed to harsh living conditions, such as poor sanitation, malnutrition, communicable diseases, and lack of integrated management of childhood illnesses (19).

Children with developmental disabilities often have complex health issues and high unmet health needs (20). As a result of caregiving responsibilities, the parents of children with developmental disabilities report lower QoL and a sense of isolation (21). Children are dependent on parents, thus, having a child with a disability has a negative impact on the parental life, as it requires many adjustments for the family members (22).

This study showed significantly lower scores in the overall QoL compared to control groups. In the four domains of QoL, significant differences were observed in the physical and environmental domains while no statistically significant differences were observed in the social and psychological domains. While the overall QoL scores were low in all domains for study and control groups, the scores were lower in the study group (*p* = *0.01*). These findings are similar to a recent study by Pecor et al. (23) who reported overall lower scores in parental QoL during the COVID-19 outbreak. The lower overall QoL in parents of children with disabilities during the COVID-19 pandemic can be attributed to increased caregiver burden. Another recent study by Shah *et al*. reported worsening of symptoms in pediatric patients with ADHD and an increase in the number of negative interactions with parents during COVID-19 (24).

The physical domain of QoL relates to physical health, sleep, pain, and coping with everyday life and daily physical activities. The mean scores in the physical domain of QoL scores were low overall; however, statistically significant differences were observed in the physical health domain of QoL between the study and control groups. The lower QoL score in the physical domain of QoL is aligned with the findings of prior studies (25–28). The overall low scores in the both study and control groups may be attributed to the ongoing COVID-19 pandemic, which has seen its second and third waves in Pakistan (29, 30). Numerous studies have explored the negative effects of the COVID-19 pandemic on the physical, social, and psychological domains of QoL (23, 31–34).

We did not find any statistically significant difference in the psychological domain of QoL between the study and control groups, which is similar to findings reported by Leung et al. (14). Other studies have reported significant differences in the psychological domain of QoL (25–28). Further research may help to uncover the underlying reasons for this observation.

The social domain of QoL is related to satisfaction with personal relationships and support structure. We did not find any statistically significant differences in the social domain of QoL; however, prior studies have found significant impairment in the social domain of QoL (25, 27, 28, 35). This is an interesting finding, with the plausible explanation being the cultural differences that exist in parental experiences toward child disability (36, 37). The absence of differences in the social domain of QoL could be explained by the Muslim values and the South Asian family-oriented structure of Pakistani society, which may serve as a source of informal support in disability. In a study exploring perceptions toward disability, Ow et al. (38) reported that 48% of Chinese mothers having a child with disabilities had at least one formal source of support; however, none of the Muslim mothers reported needing a formal source of support. Mohamed Madi et al. (36) reported that religious beliefs and cultural norms were major factors in the perceptions of mothers towards child disability in Saudi Arabia, another Muslim majority country.

The environmental domain of QoL is related to physical safety and security, home environment, and health and social care. We observed significantly lower mean scores in the environmental domain of QoL in the study group. This is similar to the findings published in several other studies (14, 25, 27, 28). The greater caregiver burden of having a child with developmental disorders, such as limited socio-adaptive functioning, difficult behaviors and limited social skills, inability to understand the condition of the child, difficulty obtaining the correct diagnosis, and stressful experiences with professionals, contributes to lower scores on the environment domain (25, 26, 28, 36). In addition, limited access to support services due to the COVID-19 pandemic and financial difficulties may further worsen the stress burden.

Prior research has identified that parental QoL is also impacted by the type and degree of disability in the child (39, 40). Disability, depending on the type of disability variable, favors parents having a child with a learning disability, followed by parents having a child with a physical disability, parents having a child with an intellectual disability, and finally parents having a child with Autism, who have the lowest degree of QoL (39).

The subset analysis based on the type and degree of disability could not be performed in this study, due to a disproportionately large sample of parents having children with ASD in the study group, which is a limitation of this study.

Further exploration of this subject with a larger sample size may reveal differences in the degree to which different developmental disabilities in the children affect parental QoL.

This study showed an overall low score in all domains, namely, physical health, environment, social relationships, and psychological health, for both groups. This might be due to the COVID-19 pandemic, and the results were consistent with many other studies done on QoL during the COVID-19 pandemic (23, 41–44). Future efforts aimed at therapeutic interventions in child disability might benefit from focusing on physical and environmental domains of QoL in addition to strengthening support structures through informal approaches.

## Conclusion

This study demonstrates statistically significant differences in the WHOQoL-BREF scores in the physical and environmental domains of parents of children with developmental disabilities during the COVID-19 pandemic. No significant differences were observed in the psychological and social domains of QoL between the study and control groups.

## Data Availability Statement

The original contributions presented in the study are included in the article/supplementary material, further inquiries can be directed to the corresponding author/s.

## Ethics Statement

The studies involving human participants were reviewed and approved by Ethics Review Committee, Aga Khan University Hospital, Karachi, ERC-2020-5476-14718. The patients/participants provided their written informed consent to participate in this study.

## Author Contributions

UA: drafting, data acquisition, interpretation, and revision. VB: design of the work, data analysis, interpretation, and drafting. NA: conception, data acquisition, interpretation, and drafting. SJ: conception, supervision of data acquisition, drafting, critical revision, and final approval. All authors contributed to the article and approved the submitted version.

## Conflict of Interest

The authors declare that the research was conducted in the absence of any commercial or financial relationships that could be construed as a potential conflict of interest.

## Publisher's Note

All claims expressed in this article are solely those of the authors and do not necessarily represent those of their affiliated organizations, or those of the publisher, the editors and the reviewers. Any product that may be evaluated in this article, or claim that may be made by its manufacturer, is not guaranteed or endorsed by the publisher.
